# Antioxidant Recovery from Massachusetts Cranberry Pomace: The Role of Solvent

**DOI:** 10.3390/antiox15060682

**Published:** 2026-05-29

**Authors:** Maureen Otieno, Elena De Pra, Ryley Thatcher, Catherine Neto

**Affiliations:** Department of Chemistry and Biochemistry and Cranberry Health Research Center, University of Massachusetts Dartmouth, North Dartmouth, MA 02747, USA; motieno1@umassd.edu (M.O.); edepra@umassd.edu (E.D.P.); rthatcher1@umassd.edu (R.T.)

**Keywords:** cranberry pomace, antioxidant activity, polyphenols, phytochemical characterization, solvent extraction

## Abstract

Cranberry pomace is a rich, but underutilized source of polyphenols and other bioactive compounds. This study assessed the efficacy of six solvent mixtures comprising combinations of ethanol, methanol, acetone, formic acid, and water for extraction of antioxidants from pomace. The antioxidant activity and total phenolic content of the cranberry pomace extracts were evaluated using DPPH (2,2-diphenyl-1-picrylhydrazyl), ABTS•^+^(2,2′-azinobis(3-ethylbenzthiazolin-6-sulfonic acid), FRAP (ferric reducing power), and Folin–Ciocâlteu assays, the total proanthocyanidin content was estimated using the dimethylaminocinnamaldehyde (DMAC) method, and individual polyphenolics and triterpenoids were determined using HPLC-DAD and UPLC-MS. Extracts exhibited a broad range of total phenolic content at 21–166 mg gallic acid equivalents (GAE)/g extract), total PACs (proanthocyanidins) at 6–240 mg PAC equivalents/g extract, anthocyanins at 0.42–1.77 mg/g extract, flavonols at 4.09–11.7 mg/g extract, and triterpenoids at 85.6–287 mg/g extract. Antioxidant activities varied widely and correlated positively with all polyphenol categories, but negatively with triterpenoids. An extract produced using an acetone, methanol, water and formic acid mixture demonstrated optimal antioxidant properties, total phenolic content, and total proanthocyanidin content and was further characterized. Our findings emphasize the importance of solvent selection for targeted bioactive constituents and highlight cranberry pomace as a promising source of antioxidants.

## 1. Introduction

In recent years, there has been a significant increase in interest in berry-derived products, primarily due to their rich array of bioactive compounds that provide numerous health benefits. Cranberries (*Vaccinium macrocarpon*) are produced and consumed worldwide in the form of whole-fruit products, beverages, and supplements. Massachusetts (MA) is a major cranberry-producing region in North America, with 2.26 million barrels produced in 2022 [1], over 90% of which is processed. Large quantities of pomace are generated as a sidestream of processing, prompting continued evaluation of this raw material as a source of bioactive secondary metabolites. Cranberry pomace, generated primarily from cranberry juice production, consists of skin, pulp residues, stems, and seeds [2]. This pomace is a rich natural source of dietary fiber (cellulose, lignin, pectin, and hemicelluloses) fat, protein, ash, moisture, and phytochemicals [2,3].

Traditionally, fruit pomaces have been used as animal feed; however, their applications may be limited due to low protein content and seasonal availability. The presence of polymeric polyphenols, such as lignin, can reduce digestibility by inhibiting cellulolytic and proteolytic enzymes, as well as the growth of rumen bacteria [4]. Cranberry pomace has also been used as fertilizer, but high levels of phenolic compounds can inhibit seed germination. Currently, most pomace is disposed of by returning it to the land, which can cause environmental stress due to its low pH [5,6]. An estimated 60–95% of cranberry fruit produced is processed for commercial use generating pomace [1,7], suggesting further exploration of its properties would be beneficial.

Cranberry fruit is a rich source of antioxidant phytochemicals [8]. We previously reported that antioxidant properties of fruit from MA and Oregon (OR) correlated positively with polyphenol content [9], and have also reported that polyphenol- and triterpenoid-rich extracts of whole cranberry fruit were capable of reducing tumor formation and inflammation in a mouse model of colitis [10]. Flavonols, proanthocyanidins (PACs), anthocyanins, and triterpenoids are concentrated in the cranberry skin [11,12], thus our interest in understanding how best to recover them from pomace. Extraction of these compounds often employs solvent systems containing methanol, acetone, ethanol, ethyl acetate, or other organic solvents combined with water and acidified to improve matrix swelling, release, and stabilization of pH-sensitive constituents such as anthocyanins [7,13]. As these phytochemical classes differ substantially in polarity and chemical structure, solvent composition can strongly influence extraction efficiency and selectivity [14]. Previous studies have investigated cranberry pomace extraction using aqueous ethanol, acetone-based systems, heat, pressurized liquid extraction, supercritical fluids, extrusion-assisted, and high-intensity ultrasound-assisted approaches [11,15,16]. For example, supercritical CO_2_ was found effective for extraction of lipophilic fatty acids, and pressurized liquid extraction using ethanol–water systems was optimized for recovery of PACs and anthocyanins [11]. Extrusion-assisted extraction at high temperatures was applied successfully to recover PAC oligomers of varying size ranges [15]. High-intensity ultrasound with natural deep eutectic solvents was found to enhance anthocyanin recovery [16]. These approaches demonstrated effective recovery of selected phytochemical classes, but require specialized equipment, elevated temperatures, or complex extraction systems that may limit scalability or risk degradation of heat-sensitive compounds.

The need remains for simple extraction strategies capable of recovering a broad spectrum of bioactive compounds or targeting specific classes using gentle, cost-effective methods for further evaluation of pomace as a source of biologically relevant phytochemicals. Limited data exist on optimization of extraction mixtures for antioxidant potential, and data on solvent efficacy for the recovery of flavonols or triterpenoids from cranberry pomace are scarce. As described herein, we addressed this gap by utilizing a range of solvent systems with gentle bath ultrasonication at temperatures below 25 °C to extract soluble secondary metabolites from cranberry pomace. Our objectives were to optimize phytochemical extraction while avoiding conditions that can degrade polyphenols, identify which mixtures maximize total phenolic content, total PACs, and other analytes of interest, and determine the relative antioxidant potentials of these preparations. The extracts were analyzed for phytochemical composition, free-radical scavenging, and ferric ion reducing abilities. Multivariate analysis was used to determine relationships between antioxidant activity and the composition of the extracts. These data may help guide recovery of natural antioxidants from cranberry pomace for further study.

## 2. Materials and Methods

### 2.1. Samples and Reagents

Cranberry pomace (lot number 10182018), provided by Ocean Spray Cranberries, Inc. (Middleborough, MA, USA), was produced from fruit of mixed cultivars (primarily Stevens and Early Black) harvested in Massachusetts in October 2018. The fresh pomace was stored frozen (−20 °C) until use. Methanol LC/MS grade, water LC/MS grade, acetonitrile LC/MS grade, formic acid LC/MS grade, and sodium carbonate were purchased from Fisher Scientific (Hampton, NH, USA). Quercetin hydrate, Folin–Ciocâlteu reagent, gallic acid, diphenyl-1-picrylhydrazyl, and 4-(dimethyl-amino)cinnamaldehyde, were purchased from Sigma Aldrich (St. Louis, MO, USA). Trolox (6-hydroxy-2,5,7,8-tetramethylchroman-2-carboxylic acid) for DPPH was purchased from EMD Millipore Corp., Burlington, MA, USA, and 4-hydroxycinnamic acid (p-coumaric acid) was purchased from Aldrich Chemical Company (Milwaukee, WI, USA). Cranberry proanthocyanidins (PACs) standard for the DMAC analysis were previously prepared from an isolated cranberry PAC fraction containing A-type flavan-3-ol oligomers, which was characterized by matrix-assisted laser desorption–ionization time-of-flight (MALDI-TOF) mass spectrometry. The myricetin standard was purchased from Indofine Chemical Company (Hillsborough Township, NJ, USA).

### 2.2. Extract Preparation

Frozen cranberry pomace was ground using a coffee grinder, then freeze-dried in a lyophilizer (LABCONCO Free Zone 4.5 L −86 °C, 0.805 mbar) for 48 h, and finally ground to a fine, dry powder. The pomace powder was extracted using a method described by Kunal Patel 2011 [17] with modifications. The sample-to-solvent ratio used was 1:10. The solvent mixtures included 40:40:1:19 acetone–methanol–formic acid–water (CPAMFAH_2_O), 70:0.1:29.9 methanol–formic acid–water (CPMFAH_2_O), 100% ethanol (CPE), 100% methanol (CPMON), and 70:0.1:29.9 ethanol–formic acid–water (CPEFAH_2_O). Briefly, 40 g of pomace powder was suspended in an extraction solvent. The mixture was stirred using a magnetic stirrer for one hour at room temperature, ultrasonicated for 30 min, and stored overnight at 4 °C. This was vacuum-filtered using a Büchner funnel and filter paper the next day, and the supernatant was collected. The solid residue was re-extracted with half the original volume of the extraction solvent, stirred for one hour at room temperature, ultrasonicated for 30 min, and refrigerated overnight, followed by vacuum filtration and a third re-extraction. The filtrates were combined, rotary-evaporated to remove the organic solvent, freeze-dried to remove excess water, and stored at −20 °C until use. A separate sample of pomace was extracted using a rapid room-temperature extraction method [18] with 100% methanol (CPM), aiming to compare the efficacy of incubating each extraction overnight vs. carrying out all extraction steps in one day. Pomace powder (40 g) was suspended in an extraction solvent. The mixture was stirred using a magnetic stirrer for one hour at room temperature and ultrasonicated for 30 min. Extractions proceeded as above, without overnight incubation.

### 2.3. Determination of Flavonols, Phenolic Acids, and Anthocyanins by HPLC-DAD

High-performance liquid chromatography with diode-array detection (HPLC-DAD) was used to identify and quantify phenolic acids, anthocyanins, and flavonols in each extract according to previously published methods, with minor modifications [9]. Analyses were performed (*n* = 4) on a Waters Alliance e2695 HPLC system at a flow rate of 0.9 mL/min, injection volume of 20 μL, using Empower 3 software, coupled with a 2998-photodiode array (PDA) detector. An Atlantis T3 column (4.6 × 150 mm, 3 μm; Waters) was used as the stationary phase. Proanthocyanidins, phenolic acids, flavonols, and anthocyanidins were detected at 280 nm, 310 nm, 355 nm, and 520 nm, respectively. The mobile phase A consisted of water–phosphoric acid (99.5:0.5, *v*/*v*), while mobile phase B was made of a mixture of water–acetonitrile–glacial acetic acid–phosphoric acid (50.0:48.5:1.0:0.5, *v*/*v*/*v*/*v*). The total running time was 31 min. The gradient elution proceeded as follows: 0–10 min (10–50% B), 10–16 min (50–60% B), 16–24 min (60–95% B), 24–28 min (95–40% B), 28–30 min (40–10% B), and 30–31 min (10% B). The extracts were dissolved in 1 mL methanol and filtered through a 0.45 mm PTFE syringe filter and thereafter placed into a newly labeled Eppendorf tube before injection. The standards were dissolved in methanol to make a stock solution of 1000 µg/mL, from which various concentrations were prepared. The polyphenols were identified by matching their HPLC peak retention time patterns and UV absorbance with those of authentic commercial standards and previously published data [9]. The diode array detector (DAD) was used to collect UV/vis spectral data while the compounds were being separated. Quantification of anthocyanin peaks was based on external standard curves for cyanidin-3-galactoside, while quantification of flavonol glycosides was based on an external standard curve for quercetin-3-galactoside (hyperoside).

### 2.4. Determination of Triterpenoids by UPLC-MS

A UPLC-MS method was used to analyze the triterpenoids in each extract in triplicate, as described by Xue et al. [12]. The method employed a Waters Xevo QTOF mass spectrometer with MassLynx software version 4.1 (Waters Corporation, Milford, MA, USA) with electrospray ionization source operating in negative ion mode, and an ACQUITY UPLC system equipped with a Waters ACQUITY UPLC BEH C18 column (Waters Corporation, Milford, MA, USA) (1.7 μm, 2.1 × 100 mm), held at a temperature of 25 °C. Isocratic elution proceeded with a mobile phase of 85:15 solvent A (0.1% formic acid in methanol) and solvent B (0.1% aqueous formic acid) at a flow rate of 0.3 mL/min. The total running time was 20 min. The effluent was monitored by ESI-MS in negative ion mode in a mass range of *m*/*z* 50−1000 using a capillary voltage of 2 kV, a sampling cone voltage of 40 V, an extraction cone voltage of 4 V, a source temperature of 100 °C, a desolvation temperature of 350 °C, a cone gas flow of 50 L/h, and a desolvation gas flow of 600 L/h. Ursolic and oleanolic acids (UA and OA) were monitored at [M-H]^−^ = 455.4. Corosolic and maslinic acids (CA and MA) were detected at [M-H]^−^ = 471.4. Cis- and trans-hydroxycinnamoyl esters of UA and OA were monitored at [M-H]^−^ = 601.4. Data analysis and quantification were performed in comparison to the commercial standards. Samples were analyzed in triplicate. Linear standard curves were prepared and analyzed each day for each triterpenoid with a concentration range of approximately 0.1–2 µg/mL for UA, 0.0375–1.2 µg/mL for OA, 0.0125–0.33 µg/mL for MA, and 0.025–0.8 µg/mL for CA. The pomace extracts were analyzed at 10 µg/mL. Analyses were conducted in triplicate, and results are reported as means ± standard deviation.

### 2.5. Method Validation for HPLC-DAD and UPLC-MS

Method performance for HPLC-DAD analyses was evaluated using external standard calibration curves prepared from authentic commercial standards. Linearity was assessed using multi-point calibration curves and showed good correlation coefficients (R^2^ > 0.99) for all quantified analytes. Precision and repeatability were evaluated using triplicate injections and expressed as relative standard deviation (RSD), which remained below 5% for major analytes. Limits of detection (LOD) and limits of quantification (LOQ) were estimated according to International Council for Harmonisation (ICH) guidelines using the standard deviation of the response and the slope of the calibration curve. Quantification was performed using external standard calibration, and all analyses were conducted in triplicate. Recovery experiments were not performed, and therefore quantitative values should be interpreted comparatively rather than as absolute recovery efficiencies. For the UPLC-MS triterpenoid method, previously reported validation parameters [19] were applied to the present analysis. Detailed validation parameters are summarized in Supplementary Table S1.

### 2.6. Determination of Total Proanthocyanidin Content by DMAC Method

Total proanthocyanidin (PAC) content was determined in triplicate for each extract using a published modification of the BL-DMAC method described by Feliciano et al. [20]. 4-Dimethylaminocinnamaldehyde (DMAC) powder (0.05 g) was dissolved in a 50 mL mixture solution consisting of ethanol–37% hydrochloric acid–distilled water (75:12.5:12.5, *v*/*v*/*v*) to yield a DMAC reagent solution. The cranberry PAC standard was used instead of procyanidin A2. A cranberry fruit PAC fraction isolated and characterized as earlier described (Patel et al., 2011) [17] was dissolved in ethanol to yield a concentration of 3 mg/mL stock standard solution. The standard stock solution was diluted with ethanol to obtain a series of different concentrations that ranged from 0.046 to 3 mg/mL for constructing the PAC standard curve.

Cranberry pomace extract or cranberry PAC standard was loaded into a 96-well plate with 5 μL aliquots in triplicate. To each well was added 65 μL PAC extraction solution. The blank for this assay contained 70 μL PAC extraction solution, which was also added to separate wells. DMAC reagent (100 μL) was quickly added to each well to reach final volumes of 170 μL. DMAC assay analysis was carried out using a 96-well microplate reader (Molecular Devices SpectraMax M5, SoftMax Pro V5). The samples were placed in the plate reader, and absorbance at 640 nm was recorded every five minutes over a 30-min period. Absorbance of each cranberry sample was compared with the PAC standard that was previously prepared from an isolated cranberry PAC fraction containing A-type flavan-3-ol oligomers as earlier described (Patel et al., 2011) [17]. PAC concentrations were calculated from the calibration curve generated using the cranberry PAC standard, *y* = *mx* + *b*, where *y* represents absorbance at 640 nm and *x* represents PAC concentration. PAC content was expressed as milligrams PAC equivalents per gram extracts, as shown in the formula below:

$$\mathrm{Total}\;\mathrm{PAC}\;\mathrm{content}\;(\frac{\mathrm{mg}}{g})=\frac{x(\frac{\mathrm{mg}}{\mathrm{mL}})}{\mathrm{Sample}\;\mathrm{concentration}\;(\frac{g}{\mathrm{mL}})}$$

where *x* is the concentration of PAC from the standard calibration curve (mg/mL), and sample concentration is the concentration of the extract.

### 2.7. Determination of Total Phenolic Content by Folin–Ciocalteu Assay

The total phenolic content (TPC) of each extract was determined in triplicate using the Folin–Ciocâlteu reagent microplate method described by Ainsworth et al. (2007) and Agbor et al. (2014), with modifications [21,22]. Briefly, 20 mg of each sample was dissolved in 2 mL ice-cold 95% MeOH and ultrasonicated for 30 min, followed by incubation for 24 h. The samples were centrifuged (13,000× *g* for 5 min at rt), and the supernatant was collected in fresh 2 mL Eppendorf tubes. Different concentrations of gallic acid (2.5–0.04 mM) were used to prepare the standard curve by taking 40 μL of gallic standard solution into 7 different 2 mL Eppendorf tubes, with the blank containing 40 μL of DI water. 800 μL of 10% (*v*/*v*) Folin–Ciocâlteu reagent (800 μL of 10% (*v*/*v*)) was added to each tube and mixed well. The tubes were allowed to stand for 5 min. This was followed by the addition of 800 μL of 700 mM sodium carbonate aqueous solution to each tube and vortexed thoroughly. The volume in each tube was made up to 2 mL with DI water (360 μL), mixed well, and the tubes were allowed to stand for 2 h. A standard or blank 200 μL sample was transferred to a clear 96-well microplate, and absorbance was measured at 765 nm against the blank using a SpectraMax 190 microplate reader (Molecular Devices, San Jose, CA, USA). The standard curve was calculated from the blank-corrected A_765_ of the gallic acid standards. The TPC was calculated from the gallic acid calibration curve and expressed as milligrams gallic acid equivalents (GAE) per gram of extract using the formula below.

$$\mathrm{TPC}=\frac{C_{0}*\mathrm{MW}}{C_{1}}$$

Here, TPC is the total phenolic content in mg GAE/g extract, C_0_ is the gallic acid concentration from the standard curve in mmoles GAE/L, MW is the molecular weight of gallic acid in mg/mmole, and C_1_ is the extract concentration in g/L.

### 2.8. Determination of Antioxidant Activity by DPPH Free Radical-Scavenging Assay

The antioxidant capacity of each pomace extract was evaluated in triplicate using a modified microplate method as described by Liu, et al., 2014 [23]. DPPH powder (5 mg) was dissolved into 50 mL of methanol to yield a 100 μg/mL concentration of DPPH stock solution. Stock solutions of crude extracts were prepared in methanol at a concentration of 250 μg/mL and diluted to various concentrations using 2-factor serial dilution. A volume of 25 μL DPPH solution (handled in the dark) was mixed with each sample (250 μg/mL, 100 μL) and added to 96-well assay plates. The blank was 100 μL of methanol, and the control contained 125 μL of DPPH methanol solution. All samples were tested in triplicate. The plate was shaken gently and placed in the dark for 30 min at room temperature. The absorbance was measured at 517 nm using a SpectraMax 190 microplate reader (Molecular Devices, San Jose, CA, USA) after 30 min of reaction. The percentage of inhibition was calculated to compare free radical-scavenging antioxidant activities of all cranberry crude extracts. Results were calculated as percentage inhibition. The formula is:

$$\%\;\mathrm{Inhibition}=\frac{A0-A1}{A0}*100$$

where A0 = the absorbance of the control with no radical scavenger (DPPH + MeOH Abs), and A1 = the absorbance of remaining DPPH^▪^ in the presence of the scavenger (sample Abs).

### 2.9. Determination of Antioxidant Activity by ABTS•+ Free Radical-Scavenging Assay

A modified 2,2′-azino-bis(3-ethylbenzothiazoline-6-sulfonic acid antioxidant) potential (ABTS•+) microplate method was used to estimate the radical-scavenging potential [24,25]. Phosphate-buffered saline (PBS) (pH 7.4, 0.01 M) was prepared by weighing 0.254 g of KH_2_PO_4_, 1.44 g Na_2_HPO_4_, 8 g NaCl, and 0.2 g KCl in 1 L of DI water. pH was adjusted to 7.4 with NaOH. ABTS stock solution (7 mM) was prepared by dissolving 0.1921 g ABTS ammonium salt (MW = 548.7 g/mol) in a 50.0 mL volumetric flask using phosphate buffer solution (pH = 7.4) to obtain a solution of concentration 7 mM. Potassium persulfate stock solution (2.45 mM) was prepared by dissolving 0.0331 g of potassium persulfate (MW = 270.32 g/mol) in a 50.0 mL volumetric flask using phosphate buffer solution (pH = 7.4) to obtain a solution with a concentration of 2.45 mM. Equal aliquots of 50.0 mL 7 mM ABTS solution and 50.0 mL 2.45 mM potassium persulfate solution were mixed, and the reagent was left in the dark for 16 h. The radical cation (ABTS•+) was further diluted with 0.01 M PBS (pH 7.4) to an absorbance of 0.70 ± 0.02 at 734 nm. Stock solutions of each crude extract were prepared in triplicate in methanol at a concentration of 250 μg/mL and diluted to various concentrations using 2-factor serial dilution, then 180 µL of the diluted ABTS•+ reagent and 20 µL of standard (Trolox) and sample dilutions were loaded in a 96-well plate (triplicate). The plate was incubated in the dark at room temperature for 30 min. Absorbance was measured at 734 nm in a plate reader. Results were calculated as percentage inhibition using the formula:

$$\%\;\mathrm{Inhibition}=\frac{A0-A1}{A0}*100$$

where A0 = the absorbance of the control with no radical scavenger (ABTS•+ + MeOH Abs), and A1 = the absorbance of the remaining ABTS•+ in the presence of the scavenger (sample).

### 2.10. Determination of Antioxidant Activity by FRAP Assay

A modified ferric reducing antioxidant power (FRAP) microplate method was used to estimate the reducing potential [24,25]. The FRAP working solution was prepared on the day of use by mixing 25 mL of acetate buffer (300 mM, pH 3.6), 2.5 mL of 10 mM TPTZ in 40 mM HCl, and 2.5 mL of 20 mM 2.5 mL FeCl_3_·6H_2_O solution, or 10:1:1 (*v*/*v*/*v*). Stock solutions of each extract were prepared in triplicate in methanol at a concentration of 250 μg/mL and diluted to various concentrations using 2-factor serial dilution, then 20 µL of sample or control (Trolox at 250 µM) was added into the wells of the 96-well plate along with 180 µL of the FRAP reagent. The blank was made of methanol and FRAP reagent (*n* = 3). The prepared samples were incubated for 30 min, after which the plate was read at 593 nm. Antioxidant reducing activity was evaluated by monitoring the reduction of Fe^3+^-TPTZ to Fe^2+^-TPTZ at 593 nm. IC_50_ values were determined using OriginLab 2025 software.

$$\%\;\mathrm{Reduction}\;\mathrm{of}\;{\mathrm{Fe}}^{3+}=\frac{\mathrm{Sample}}{\mathrm{Control}}*100$$

### 2.11. Statistical Analysis

All measurements were performed in triplicate for each prepared extract, except for total flavonols, total anthocyanins, and phenolic acids, which were analyzed in four replicates (*n* = 4). Due to the limited availability of raw materials, each extraction was performed only once per solvent system; therefore, the data represent analytical replicates rather than independent extraction replicates. Results are presented as means ± standard deviation. Statistical comparisons among solvent systems were performed separately for each measured parameter. When homogeneity of variance was met, a one-way analysis of variance (ANOVA) was followed by Tukey’s multiple-comparison test. When variances were unequal, Brown–Forsythe–Welch ANOVA was followed by Dunnett’s T3 multiple-comparison test. Significance was determined using multiplicity-adjusted *p* values, with *p* < 0.05 considered statistically significant. Multivariate analyses, including principal component analysis (PCA), hierarchical clustering, and Pearson correlation analysis, were conducted in R (R Foundation for Statistical Computing, Vienna, Austria) to evaluate relationships between extract composition and antioxidant activity. Additional analyses were performed using Microsoft Excel and GraphPad Prism version 8.4.2.

## 3. Results

### 3.1. Extraction Yield

In the current study, cranberry pomace was subjected to extraction using six different solvent mixtures, resulting in extraction yields that varied between 4.6% and 9.2% (Table 1). A comparison of the extraction yields among the various aqueous solvents demonstrated that the yield for CPAMFAH_2_O (8.3%) was nearly double that of CPMFAH_2_O (4.6%) and CPEFAH_2_O (4.6%). This observation suggested that the incorporation of acetone in the extraction mixture significantly enhanced the solubility of phenolic compounds. Aqueous acetone has been shown to be a particularly effective solvent for extracting higher-molecular-weight phenolic compounds from fruit pomaces like grape pomace seeds [26,27] and other plant materials, like peach fruit [13,28].

### 3.2. Identification and Quantification of Flavonols, Anthocyanins, Phenolic Acids

HPLC-DAD analysis was employed to determine the content of individual flavonols, anthocyanins, and phenolic acids in the cranberry pomace extracts (Table 2). Method validation data appear in Table S1. Total flavonol and total anthocyanin content is also summarized in Table 2 and Figure S1. The cranberry pomace ethanol, formic acid, and water extract (CPEFAH_2_O) exhibited the highest concentrations of the three flavonol aglycones (quercetin (4.58 ± 0.01 mg/g of extract), myricetin (1.21 ± 0.001 mg/g of extract), and isorhamnetin (0.76 ± 0.01 mg/g of extract)), with CPMON displaying comparable levels. Notably, the aglycone forms of flavonols were more prevalent than their glycoside counterparts, with CPEFAH_2_O also yielding the highest glycoside content. This pattern may be attributed to ethanol’s enhanced efficiency in extracting glycosides, catechols, and tannins [13]. While CPMFAH_2_O and CPMON exhibited similar aglycone concentrations, the glycoside content in CPMFAH_2_O surpassed that of CPMON. This observation likely reflects the higher water-solubility of glycosides, resulting in their preferential extraction into the aqueous phase of the solvent system.

P-coumaric acid was identified as the most abundant phenolic acid, with CPMFAH_2_O exhibiting the highest concentration (1.67 ± 0.17 mg/g of extract), followed closely by CPEFAH_2_O (1.66 ± 0.03 mg/g of extract). Caffeic acid and chlorogenic acid were less abundant. Phenolic acid content decreased in the order CPMFAH_2_O > CPEFAH_2_O > CPMON > CPAMFAH_2_O > CPE > CPM, supporting the efficacy of methanol for extraction of phenolic acids [13]. Six anthocyanins were identified, as discussed in Section 3.2.1. Cyanidin and peonidin arabinosides were the most abundant across the extracts. Total anthocyanin content was higher for the methanolic extracts, with CPMFAH_2_O displaying the highest concentration (1.77 ± 0.13 mg/g), whereas CPE (0.42 ± 0.001 mg/g) presented the lowest. A decreasing trend in total anthocyanin content was observed in the following order: CPMFAH_2_O > CPMON > CPAMFAH_2_O > CPM > CPEFAH_2_O > CPE.

#### 3.2.1. Polyphenol Characterization of Pomace CPAMFAH_2_O

**Figure 1:**
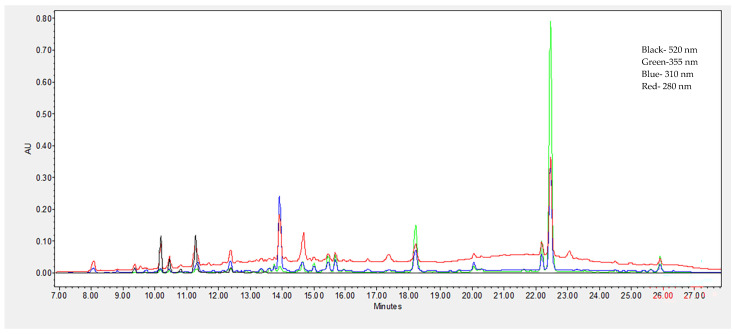
HPLC chromatogram of CPAMFAH_2_O extract at 10 mg/mL. Proanthocyanidins, phenolic acids, flavonols, and anthocyanins were monitored at 280, 310, 355, and 520 nm, respectively. Identified analyte peaks are listed in Tables S2 and S3.

Characterization of CPAMFAH_2_O was conducted by HPLC-DAD, monitoring four wavelengths: proanthocyanidins at 280 nm, phenolic acids at 310 nm, flavonols at 355 nm, and anthocyanins at 520 nm (Figure 1). The peak at retention time of 22.5 min corresponds to the major flavonol in cranberry, quercetin. The baseline observed at 280 nm exhibits a non-linear characteristic, which is often observed in cranberry-derived materials due to the variation in size and linkage among cranberry proanthocyanidins, which typically do not resolve well on a C18 column. Reverse-phase chromatography is better suited to resolution of other phenolics and flavonoids in cranberry [9], as shown in Figures S2–S4. We identified 11 anthocyanin peaks (see Figure S2 and Table S2) based on commercial standards alongside published literature [9]: 1 = cyanidin-3-O-galactoside, 2 = cyanidin-3-O-glucoside, 3 = cyanidin-3-O-arabinoside, 4 = peonidin-3-O-galactoside, 5 = peonidin-3-O-glucoside, 6 = peonidin-3-O-arabinoside. The anthocyanins were quantified based on idaein chloride (cyanidin-3-O-galactoside chloride) standard, and the profile was consistent with anthocyanins previously identified in fresh cranberries [9].

We identified 14 flavonol peaks (see Figure S3 and Table S3) by matching their retention time and UV absorbance patterns to known standards. Peaks lacking standards were tentatively identified through chromatographic data from previous reports on cranberry (Figure S3) [2]. Quercetin aglycone was the most abundant flavonol in CPAMFAH_2_O extract (2.74 mg/g), followed by myricetin aglycone (0.85 mg/g). The flavonol profile resembles that described by White et al., who employed a solvent system of acetone, water, and acetic acid (70:29.5:0.5 *v*/*v*/*v*) with extrusion for polyphenolic extraction from cranberry pomace [2]. Phenolic acids reported in cranberry are primarily derivatives of benzoic and hydroxycinnamic acids and serve as significant natural compounds utilized in food as antioxidants. Based on the available standards, we were able to identify three phenolic acids—p-coumaric acid, caffeic acid and chlorogenic acid (CGA)—as illustrated in Figure S4. Previous studies have identified the presence of gallic acid, ellagic acid, resveratrol, and p-hydroxybenzoic acid in cranberry pomace [29]. However, these were not detected in our extract. It is likely that many organic acids are released during juice extraction, resulting in their limited presence in the pomace.

### 3.3. Identification and Quantification of Triterpenoid Content

The four major triterpenoid acids identified in cranberry pomace extracts are ursolic acid (UA), oleanolic acid (OA), corosolic acid (CA), and maslinic acid (MA), as well as the hydroxycinnamoyl esters of UA and OA (Table 2). The limits of quantification (LOQ) for ursolic acid, oleanolic acid, maslinic acid, corosolic acid, and betulinic acid were 0.0625 ppm, 0.0375 ppm, 0.01 ppm, 0.025 ppm, and 0.01 ppm, respectively, while the corresponding limits of detection (LOD) were 0.0187 ppm, 0.01125 ppm, 0.003 ppm, 0.0075 ppm, and 0.003 ppm, respectively [19]. Extraction efficiency of triterpenoid acids varied widely among methods: of the six pomace extracts, CPMON exhibited the highest total triterpenoid content (287 ± 12 mg/g of extract), while CPMFAH_2_O demonstrated the lowest levels (85.6 ± 2.3 mg/g of extract). Total triterpenoid content decreased in the order CPMON > CPE > CPM > CPEFAH_2_O > CPAMFAH_2_O > CPMFAH_2_O (see Table 2), an indication that the triterpenoids were more efficiently extracted in the pure organic solvents compared to the aqueous organic solvents. UA and OA were the most abundant triterpenoid acids, while CA and MA were considerably lower. Triterpenoid ester content demonstrated a different trend, with CPEFAH_2_O (90.2 ± 5.1 mg/g of extract) being the most abundant followed closely by CPMON (89.0 ± 7.6 mg/g of extract). Total triterpenoid HCA esters decreased in the order CPEFAH_2_O > CPMON > CPM > CPAMFAH_2_O > CPE > CPMFAH_2_O.

### 3.4. Determination of Total Proanthocyanidins (PACs)

The total proanthocyanidin (PAC) content in pomace extracts varied depending on the extraction solvent used, with content ranging from 6.0 to 240 mg PAC equivalents/g of extract. Among the array of solvents assessed, the mixture of acetone–methanol–formic acid–water (CPAMFAH_2_O) yielded the highest PAC content (240 mg PAC equivalents/g of extract), indicating robust extraction efficiency for proanthocyanidins. In contrast, 100% ethanol exhibited the lowest PAC levels, suggesting its inadequacy for maximizing their extraction (see Table 2). When ethanol was combined with formic acid and water, the output increased, but it was still significantly lower than CPAMFAH_2_O.

### 3.5. Total Phenolic Content

The assessment of total phenolic content (TPC) for the pomace extracts was conducted using the Folin–Ciocâlteu method. Values ranged from a low of 21.0 mg GAE/g of extract for CPE to a high of 166.0 mg GAE/g of extract for CPAMFAH_2_O (see Figure S5 and Table 2). Extracts prepared with acidic aqueous organic solvents exhibited a significantly higher TPC than those prepared with pure organic solvents. This suggests enhanced phenolic extraction potential in aqueous acidic systems, as the hydrophilic and acidic nature of polyphenols promotes better solubility and stability at low pH.

### 3.6. Antioxidant Capacity

The antioxidant activity of the pomace extract was assessed through the application of three assays: FRAP (ferric reducing antioxidant power), DPPH (2,2-diphenyl-1-picrylhydrazyl), and ABTS (2,2′-azinobis(3-ethylbenzothiazoline-6-sulfonic acid)). To assess the relative effectiveness of the extracts, the assays were carried out using extract concentrations of 250–15.6 μg/mL, and IC_50_ values were determined utilizing OriginLab 2025 software. The IC_50_ value of a compound is inversely related to its antioxidant capacity, representing the concentration required to scavenge 50% of free radicals (DPPH/ABTS•+) or reduce the Fe^3+^ to Fe^2+^, derived from the dose–response curve. Consequently, lower IC_50_ values indicate higher antioxidant activity, while higher values signify reduced activity. Among the various extracts analyzed, those including water and formic acid displayed the strongest antioxidant activity (see Table 3), with CPAMFAH_2_O leading with the lowest IC_50_ across the three antioxidant assays: DPPH (6.46 ± 2.8 µg/mL), FRAP (2.6 ± 0.1 µg/mL), and ABTS•+ (3.7 ± 0.6 µg/mL). These data align closely with the results from the total phenolic content (TPC) analysis, reinforcing the association between phenolic compounds and antioxidant activity.

### 3.7. Principal Component Analysis

**Figure 2:**
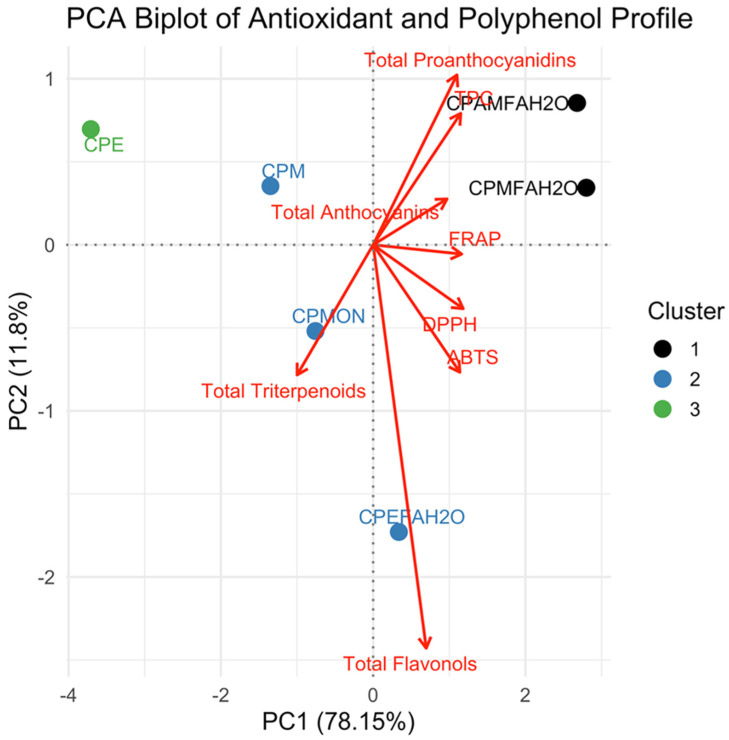
Principal component analysis biplot showing separation of cranberry pomace extracts based on phytochemical composition and antioxidant activity parameters. Vectors represent variable loadings, and clustering indicates similarity among extracts.

Multivariate analysis using principal component analysis (PCA) was performed to evaluate the relationship between the polyphenolic composition and the antioxidant activity of the extracts, considering eight chemical variables (TPC, DPPH, FRAP, ABTS•+, total proanthocyanidins, total flavonols, total anthocyanins, and total triterpenoids). IC_50_ values (DPPH, ABTS•+, and FRAP) were mathematically inverted before analysis to ensure that higher values corresponded to greater antioxidant activity. PC1 explained 78.1% of the total variance, while PC2 explained 11.8%**,** accounting for a cumulative variance of 89.9%**.** This indicates that nearly 90% of the biochemical variation among extracts can be represented in a two-dimensional space (Figure 2). Top contributors to PC1 were DPPH, FRAP, TPC, ABTS•+, and total proanthocyanidins, all with high positive loadings. Total triterpenoids had a strong negative loading on PC1 (Figure 2 and Table 4). Top contributors to PC2 were total flavonols (strong negative loading, −2.429) and total proanthocyanidins (moderate positive loading, +1.023). Total anthocyanins contributed minimally to PC2 (+0.276) (See Table 4). The PCA biplot demonstrated clear separation among extracts based on their phytochemical and antioxidant profiles. PC1 was the dominant axis of variation and largely distinguished extracts with high antioxidant and phenolic content from those with comparatively lower values.

**Figure 3:**
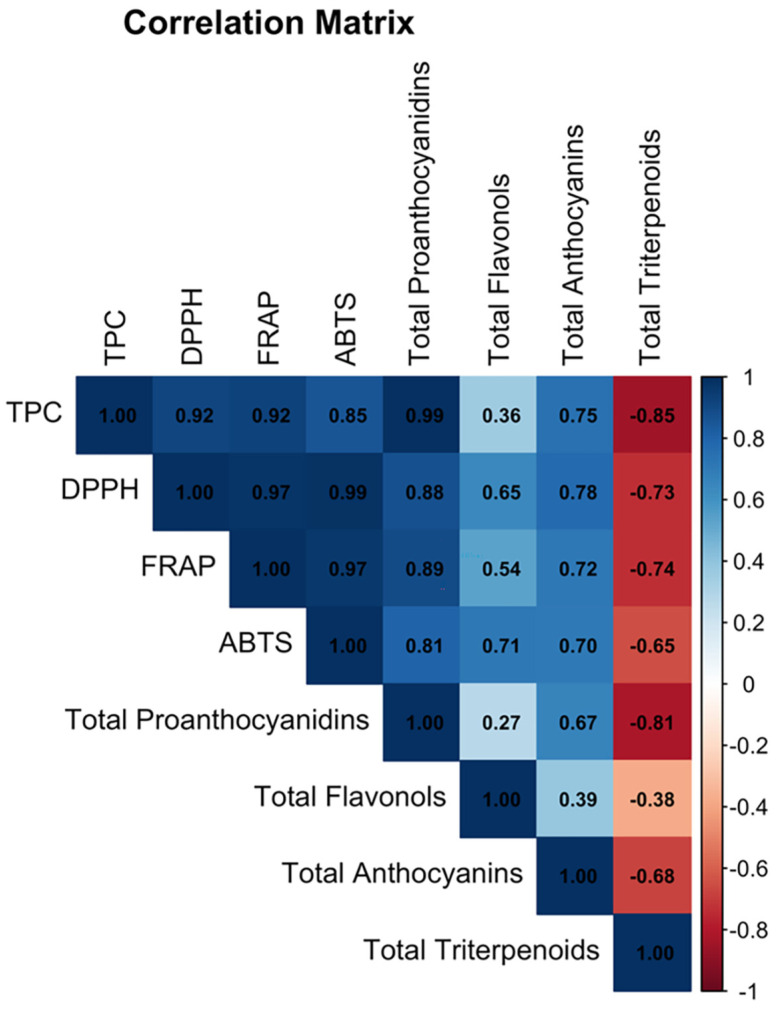
Heatmap showing Pearson correlation coefficients (r) between antioxidant activity and phytochemical variables in cranberry pomace extracts. Correlation values are in Table 6. DPPH, ABTS, and FRAP data is based on inverse IC_50_ values, where higher values correspond to stronger antioxidant activity. Positive correlations are shown in blue and negative correlations in red.

Hierarchical clustering divided the extracts into three clusters (Figure 2). Cluster 1 (CPAMFAH_2_O, CPMFAH_2_O) exhibited the highest PC1 scores, indicating the strongest antioxidant activity. Cluster 2 (CPEFAH_2_O, CPMON, CPM) displayed intermediate activity, and Cluster 3 (CPE) showed the lowest activity. Ranking by mean antioxidant activity, calculated from inverse IC_50_ values for DPPH, FRAP, and ABTS•+, indicated that CPAMFAH_2_O had the strongest activity followed by CPMFAH_2_O, whereas CPE exhibited the weakest activity. Pearson correlation analysis using mean antioxidant activity as the response variable revealed strong positive correlations with DPPH (r = 0.998, *p* < 0.001), ABTS•+ (r = 0.994, *p* < 0.001), FRAP (r = 0.979, *p* = 0.001), and TPC (r = 0.899, *p* = 0.015) (Table 5). Total proanthocyanidins also demonstrated a moderate positive correlation (r = 0.857, *p* = 0.029) with mean antioxidant activity. In contrast, total flavonols, total anthocyanins, and total triterpenoids exhibited weaker, non-significant relationships. Total triterpenoids were negatively correlated with mean antioxidant activity (r = −0.705, *p* = 0.118), suggesting limited contribution to the antioxidant assays evaluated. Pairwise Pearson correlation analysis further demonstrated strong positive associations among antioxidant assays and phenolic-rich phytochemical classes (See Table 6 and Figure 3). The strongest associations were observed between total phenolic content and antioxidant activity based on inverse IC_50_ values for DPPH, ABTS•+, and FRAP, with correlation coefficients of 0.92, 0.85, and 0.92, respectively (*p* < 0.05). Total proanthocyanidins were also moderately correlated with antioxidant activity, as indicated by r values of 0.88, 0.81, and 0.89 (*p* < 0.05) for the respective assays. Conversely, total triterpenoids exhibited negative, but statistically non-significant correlations with antioxidant activity based on inverse IC_50_ values for DPPH, ABTS•+, and FRAP (r = −0.73, −0.65, and −0.74; *p* > 0.05). Anthocyanins showed weak, non-significant positive correlations (r = 0.78, 0.70, and 0.72; *p* > 0.05) with antioxidant activity, likely due to their low abundance (<0.2%) relative to other analytes. Similarly, total flavonols demonstrated weak, non-significant positive correlations (r = 0.65, 0.71, and 0.54; *p* > 0.05) across the three assays.

## 4. Discussion

The North American cranberry (*V. macrocarpon*) fruit has value for the food industry, owing to its rich phytochemical content. The juice extraction process leads to pomace as a sidestream that can still retain substantial amounts of biologically active components that may be reused as a functional raw material [30]. The recovery of phenolic compounds from fruits has frequently employed liquid extraction techniques and varying temperatures. Aqueous mixtures containing ethanol, methanol, and acetone are often recognized for their efficacy in isolating phenolics from fruits for the study of biochemical properties and other potential applications [13]. In this study, pomace was extracted using six different solvent mixtures and mild ultrasonication. Heat was avoided to preserve sensitive flavonoids. 100% ethanol (CPE) with overnight extraction at 4 °C produced the highest extraction yield on a weight basis at 9.2%. Other mixtures yielded lower recoveries (Table 1), but were more effective in targeting phenolics and optimizing antioxidant activity. This is likely due to factors such as solvent polarities, variability in pH, and the structural diversity of pomace constituents, each of which affects overall extraction efficiency [31].

*Flavonols.* Cranberries are rich in three types of flavonoids: flavonols, anthocyanins, and proanthocyanidins (PACs) [32]. Flavonols are characterized by a 3-hydroxyflavone backbone [33] and known to be concentrated mainly in the skin of fruits. Approximately 75% of the cranberry flavonols are quercetin glycosides, with quercetin 3-O-galactoside being the predominant form in fruit [34]. Quercetin was most abundant of the flavonols in pomace extracts (Table 2), consistent with a report on aqueous ethanol extracts that found quercetin 10–17 times more abundant than its glycosides [32]. HPLC analysis detected 14 flavonols, consistent with a previous study using extrusion at temperatures of 150–190 °C followed by acetone–water–acetic acid (70:29.5:0.5 *v*/*v*/*v*) to extract pomace extrudates [15]. We found acidified aqueous ethanol (CPEFAH_2_O) the most effective solvent mixture for extracting flavonols from freeze-dried, ground pomace at room temperature via gentle sonication. CPEFAH_2_O contained 4.58 mg quercetin/g extract, 1.21 mg myricetin/g extract, and 0.76 mg isorhamnetin per g extract, as well as the highest total flavonol content of 11.7 ± 0.03 mg flavonols/g extract. Previous research indicates that cranberry flavonols possess antioxidant [35,36] and cardiovascular system-enhancing effects [37]. Anti-proliferative properties of quercetin against various cancers in vitro and in vivo are have been [34], which can depend on the form of quercetin [33]. Murphy et al. reported that quercetin was able to inhibit the growth of the MCF-7 human breast adenocarcinoma, HT-29 human colon adenocarcinoma, and K562 human chronic myelogenous leukemia cell lines with GI_50_ values in the range of 15–60 mg/L [34,38]. Myricetin reportedly induces apoptosis in colon cancer cells by increasing the expression of nucleoside diphosphate kinase and other caspase-regulated apoptotic proteins [33,39].

*Anthocyanins.* Solvent efficacy differed between flavonols and anthocyanins. CPMFAH_2_O exhibited the highest total anthocyanin content at 1.77 ± 0.13 mg/g extract, followed by CPMON at 1.39 ± 0.01 mg/g and CPAMFAH_2_O at 1.33 ± 0.02 mg/g. Anthocyanins are widely distributed in fruits, flowers, and grains and are responsible for intense red, orange, blue, and purple colors. They function to prevent the production of damaging free radicals and shield leaves, flowers, and fruits from ultraviolet light. Anthocyanins are prone to degradation by various external environmental conditions, including temperature, pH, oxygen, light, and enzymes [40,41]. In studies of other berries, acidified methanol yielded the best results for anthocyanin content from blackberries [42]. Methanol has also been reported for superior extraction efficiency for phenolic acids and catechin [13]. White et al. identified six anthocyanin glycosides in cranberry pomace extract prepared using acetone–water–acetic acid (70:29.5:0.5 *v*/*v*/*v*), for a total of 121.4 ± 5.9 mg/100 g of DW anthocyanins (or 1.21 mg/g DW) [2]. Our extracts ranged from 1.77 mg/g of extract for CPMFAH_2_O to a low of 0.42 mg/g for CPE (Table 2). HPLC analysis found the arabinosides were the most plentiful, with peonidin arabinoside being the most abundant in the CPAMFAH_2_O pomace extract. Cranberry anthocyanins have demonstrated antioxidant, metabolic, gut health, and other properties that may improve overall health [43]. Yan et al. reported that cyanidin-3-galactoside extracted from cranberry fruit effectively scavenges free radicals and inhibits the oxidation of low-density lipoproteins [35]. Ho et al. conducted studies on mice, revealing that peonidin-3-glucoside significantly reduced the metastasis of lung carcinoma cells [44]. A glycemia-lowering effect is also associated with cyanidin-3-glucoside [45]. Metagenomic analysis revealed that supplementation of mice with blueberry and cranberry anthocyanin extracts led to a reduction in plasma lipopolysaccharide levels linked to a diminished relative abundance of *Rikenella* and *Rikenellaceae* in the gut and enhanced growth of genera such as *Lachnoclostridium*, *Roseburia*, and the *Clostridium innocuum* group, resulting in an increased production of fecal short-chain fatty acids (SCFA) [46].

*Phenolic acids.* Cranberries contain both hydroxybenzoic and hydroxycinnamic acids (HCAs). They are rich in benzoic acid with lower levels of 2,4-dihydroxybenzoic acid, p-hydroxybenzoic acid, and o-hydroxybenzoic acid. HPLC analysis showed that some hydroxycinnamic acids are retained in pomace after juicing, with p-coumaric acid the most abundant phenolic acid in our pomace extracts. HCAs are also found esterified to UA and OA in pomace extracts. Caffeic and chlorogenic acids were present at lower concentrations, consistent with reports on the fruit [47]. Hydroxybenzoic acid and gallic acid were not detected in our extracts. Extraction mixtures incorporating water and formic acid were more effective at extracting caffeic, coumaric, and chlorogenic acids, especially CPMFAH_2_O. Phenolic acids such as p-coumaric acid contribute to cranberries’ overall antioxidant capacity by scavenging free radicals and supporting cellular health [48]. Its ability to act as an antioxidant has been shown in cultured endothelial cells subjected to elevated glucose and free fatty acid levels in keratinocytes exposed to UV radiation and in lens epithelial cells treated with hydrogen peroxide [49].

*Triterpenoids.* Cranberry fruit and pomace were previously reported as an abundant source of triterpenoid acids with potential anti-inflammatory properties [12,50]. Ursolic acid (UA) is the most abundant pentacyclic triterpenoid reported in pomace, followed by oleanolic acid (OA) [12]. Here, we found 100% ethanol (CPE) the most effective in producing a triterpenoid-rich extract with a UA content of 122 ± 1.8 mg/g. Most extracts also contained abundant HCA esters of UA and OA, with CPE the highest at 90.2 ± 5.1 mg/g total esters. The trend in triterpenoid content among extracts (Table 2), where extraction mixtures incorporating water yield the lowest, is likely due to their poor water-solubility. Less polar mixtures were better able to extract the nonpolar triterpenoids compared to the more polar polyphenols. Cranberry triterpenoids may be present in food products and some dietary supplements made from whole cranberry fruit [51], although the content varies widely among commercial supplements [52]. UA and its HCA esters have demonstrated anti-inflammatory properties as well as the ability to inhibit the proliferation of colon, liver, and breast cancer cells [53,54,55]. UA was also a major component of a nonpolar cranberry extract that reduced inflammation and tumor metrics in a mouse colitis model [10].

*PACs.* Cranberry PACs, polyflavan-3-ol oligomers connected through A-type and B-type interflavan carbon bonds [32], are known to inhibit the adhesion of p-fimbriated *Escherichia coli* to the urothelial cells lining the bladder. Hence, cranberry products have been widely used for several decades to prevent urinary tract infections (UTIs) [56]. Most commercial extracts are standardized to 15% and 33% PACs [57,58]. Cranberries at 100 g fresh weight (FW) provide 419 ± 75 mg total flavan-3-ols of varying degrees of polymerization (DP) [59]. They are by far the most plentiful class of polyphenols in cranberry fruit. We found that PACs were most efficiently extracted using CPAMFAH_2_O, with 240 mg PAC equivalents/g of extract. This could be attributed to including 40% acetone, which has been shown to be the best solvent for extracting high-molecular-weight phenolic compounds that methanol might not extract [13]. Klavins et al. reported optimal extraction of PACs from cranberry press residues using acetone at a concentration of 53% without the addition of an acidifying agent [24]. The non-aqueous solvents were much less effective at extracting PACs. The inclusion of aqueous acidic media may better facilitate the release of the larger oligomers from plant material. Due to instrument limitations and the complexity due to varied DP [36], the isolation and identification of individual proanthocyanidins was outside the scope of this study. Further analysis of pomace extracts by MALDI-TOF-MS, a method of choice for PACs, is recommended [32].

*Total phenolics.* The Folin–Ciocâlteu (F–C) assay is commonly used to compare the total phenolic content in various plant or food samples [59]. Despite its popularity, the F–C assay is not limited to estimation of phenolic compounds, as the reagent can be reduced by other nonphenolic compounds such as vitamin C, sugars, and amino acids that may be present [21]. As polyphenols differ considerably in their quantitative response to the F–C reagent, the overall composition and the standard used for quantification will influence estimated polyphenol content [60], though it is useful for comparing samples from a single source. Here, CPAMFAH_2_O was the most effective mixture at maximizing total phenolic content (TPC) from cranberry pomace, with the trend being CPAMFAH_2_O > CPMFAH_2_O > CPEFAH_2_O > CPMON > CPM > CPE. The efficiency of CPAMFAH_2_O may be due to acetone’s ability to extract high-molecular-weight compounds such as PACs despite its low relative polarity (0.355), while more polar methanol (0.762) more effectively extract phenolic acids and anthocyanins. Sufficient water is included to swell the pomace. Extraction solvents incorporating higher water content may retain more non-phenolic compounds, such as carbohydrates, as previously suggested [61]. Previous fruit pomace extraction studies demonstrate that the solvent efficacy for polyphenols varies depending on the extraction method and type of pomace. Tamkute and coworkers reported that pressurized liquid extraction (PLE) of cranberry pomace with water was more effective than PLE with ethanol or Soxhlet extraction in maximizing total phenolic content and antioxidant activities [11]. Previous studies on extraction of polyphenols from grape pomace found 50/50 water–ethanol or water–acetone the most effective solvents for extraction of natural phenolic compounds [62], and aqueous acetone solutions overall performed better than other organics [26]. Higher TPC values when using (70/30 *v*/*v*) aqueous acetone vs. (70/30 *v*/*v*) aqueous methanol for blackberry phenols have been reported, as well as higher antioxidant capacities [42]. For cranberry pomace, we found that combining both acetone and methanol with water in acidic media was the most effective approach.

*Antioxidant activity.* To compare antioxidant properties, at least two methods is recommended [42]. The free radical-scavenging antioxidant activity of the pomace extracts was determined using DPPH and ABTS•+ methods, and the ferric reducing antioxidant power was determined using the FRAP assay. These tests can be influenced by factors such as the duration of the reaction and the concentration of the sample [63], but all have been widely used to assess the antioxidant capacity of various plant extracts and natural products due to their rapidity, reliability, and simplicity [64]. Both ABTS•+ and DPPH are versatile radical-scavenging assays for hydrophilic and organic-soluble antioxidants [65,66,67]. Similar trends were found with the two free radical-scavenging assays. Organic solvent mixtures enriched with water and formic acid yielded the best DPPH radical-scavenging activity (Table 3), following the trend CPAMFAH_2_O > CPMFAH_2_O > CPEFAH_2_O > CPMON > CPM > CPE. ABTS•+ radical-scavenging data exhibited a similar pattern, with CPAMFAH_2_O demonstrating significantly higher antioxidant activity (IC_50_ = 3.70 µg/mL). CPMFAH_2_O (IC_50_ = 8.95 µg/mL) and CPEFAH_2_O (10.7 µg/mL) were also effective.

The ferric reducing power of the pomace extracts demonstrated a similar trend to the DPPH radical-scavenging activity, with CPAMFAH_2_O exhibiting the highest activity and ethanol ranking last. The FRAP test is a conventional method based on single electron transfer (SET) that measures the reduction of ferric ion (Fe^3+^)–ligand complexes to the intensely blue ferrous complex (Fe^2+^) by antioxidants under acidic conditions (pH = 3.6) [63]. IC_50_ values for the FRAP reaction were lower compared to those of radical-scavenging assays, ranging from CPAMFAH_2_O at 2.61 µg/mL to CPE at 14.9 µg/mL. The low IC_50_ values support the strong potential of cranberry pomace polyphenols to reduce potentially harmful ferric ions. Inclusion of 40% acetone, 40% methanol, and 20% aqueous formic acid in CPAMFAH_2_O yielded the highest TPC, total PACs, and overall antioxidant activity. In a previous cranberry pomace study using water–acetone mixtures with or without acid, 53% acetone with water was found most effective in extraction of PACs [24], and this strongly correlated with antioxidant properties.

*Correlation analysis.* Multivariate analysis with Pearson correlations (Table 5 and Table 6; Figure 3) provides further insight into the relationships between extract composition and antioxidant properties. The strong positive correlations observed between antioxidant activity and total phenolic content in pomace extracts suggest that total phenolic content can serve as a reliable predictor of antioxidant activity. The data also indicate that various phenolic classes found in cranberry pomace, particularly the PACs, significantly contribute to free-radical scavenging and antioxidant reduction potential through mechanisms such as hydrogen atom donation and electron transfer. Variation among the three antioxidant assays may also be attributed to the distinct mechanisms of action inherent to each assay, underscoring the complexity of antioxidant interactions. DPPH primarily measures hydrogen atom donation (HAT), a process in which anthocyanins and flavonols excel. FRAP measures electron transfer (ET), and while anthocyanins and flavonols can participate in ET, their primary scavenging mechanisms may not be as effectively represented in FRAP. The ability of anthocyanins and flavonols to donate hydrogen atoms might contribute significantly to their effectiveness in the DPPH assay compared to others [68]. Reports on antioxidant activity of cranberry fruits grown in the U.S. or Poland have found strong correlations with total phenolic, flavonoid, and proanthocyanidin content [9,69]. We observed the same for our pomace extracts. However, correlation with anthocyanins was not statistically significant, possibly due to their low abundance compared to the other polyphenols. Anthocyanins are highly water-soluble, and the majority are likely removed with the juice in processing.

Principal component analysis indicated that total phenolics and proanthocyanidins were the primary drivers of antioxidant variation among the extracts. The alignment of these variables along PC1, which explained most of the variance (78.1%), suggests that high concentrations of these compounds are associated with stronger antioxidant activity. The minor contribution of flavonols and anthocyanins to PC2 reflects secondary compositional differences that may modulate antioxidant behavior. Hierarchical clustering corroborated the PCA findings: extracts with high total phenolics and proanthocyanidins (Cluster 1) show superior antioxidant activity. The lower activity of CPE (Cluster 3) corresponded to both lower phenolic content and a relatively higher triterpenoid concentration, which negatively correlated with antioxidant activity. Correlation analysis confirmed the phytochemical basis of the observed activities. Strong positive correlations between TPC, proanthocyanidins, and antioxidant assays indicated that polyphenolic composition is the major determinant of radical-scavenging activity. The negative correlation of total triterpenoids with antioxidant activity suggests that their abundance does not contribute to and may even dilute overall antioxidant potency in these extracts. Overall, the classification of extracts is guided by a combination of antioxidant performance and phytochemical composition, with PCA and hierarchical clustering providing complementary evidence. These results support the use of CPAMFAH_2_O or CPMFAH_2_O solvent combinations to maximize the extraction of cranberry pomace antioxidants.

*Solvent specificity*. The present findings demonstrate that no single solvent system was optimal for all compound classes measured in cranberry pomace. Under the evaluated extraction conditions, CPAMFAH_2_O was most effective for recovering total phenolics and proanthocyanidins and produced the strongest antioxidant activity, whereas CPMFAH_2_O favored anthocyanin and phenolic acid recovery, CPEFAH_2_O produced the highest total flavonol content, and CPMON/CPE favored extraction of less polar triterpenoids. Thus, solvent selection should be guided by the targeted phytochemical class for specific applications. However, the biological efficacy, extract stability, and practical applicability of these extracts in food systems remain to be established.

The limitations of this small-scale study should be acknowledged. Cranberry pomace was obtained from a single production source and harvest season; therefore, compositional variability associated with cultivar, growing region, or processing conditions was outside its scope. Triplicate measurements represent analytical replicates rather than fully independent extraction replicates; therefore, extraction reproducibility will require further study. As the number of extracts analyzed was small, multivariate analyses (PCA and correlation analysis) should be interpreted as exploratory. Finally, the methanol- and acetone-containing solvent systems demonstrated high extraction efficiency, but these solvents are not directly applicable to food-grade commercial formulations without additional validation of solvent removal, safety assessment, and process optimization. Future studies evaluating extract stability, sensory properties, bioavailability, residual solvent content, and practical food application performance are recommended.

## 5. Conclusions

In the present study, six solvent mixtures were compared for the extraction of antioxidant-associated phytochemicals from cranberry pomace. The antioxidant activity was assessed using DPPH, ABTS•+, and FRAP assays. Validated HPLC-DAD and UPLC-MS methods were applied to determine the composition of flavonols, anthocyanins, phenolic acids, and triterpenoids in each extract, and multivariate analysis provided correlations between composition metrics and antioxidant potential. Correlation analysis indicated that total phenolic content and total proanthocyanidins were most strongly associated with radical-scavenging and reducing capacity in cranberry pomace extracts. In contrast, total triterpenoids were negatively associated with antioxidant activity, indicating that triterpenoids did not contribute substantially to radical scavenging or ferric reducing power. CPAMFAH_2_O was the most effective solvent system overall under the tested conditions, producing the highest recovery of total phenolics and proanthocyanidins and the strongest antioxidant activity. Further testing would be needed to determine the suitability of this method for food-based applications. These findings demonstrate that solvent composition is a key determinant of phytochemical recovery and antioxidant activity in cranberry pomace extracts.

## Figures and Tables

**Table 1:** Extraction yield of cranberry pomace extracts prepared using different solvent systems.

Solvent	Pomace (g DW)	Extract Net Weight (g)	% Recovery
CPAMFAH_2_O (40/40/1/19 *v*/*v*/*v*)	40	3.30	8.3
CPMFAH_2_O (70/0.1/29.9 *v*/*v*/*v*)	40	1.85	4.6
CPM (100% methanol)	40	3.55	8.9
CPMON (100% methanol)	40	2.85	7.1
CPEFAH_2_O (70/0.1/29.9 *v*/*v*/*v*)	40	1.84	4.6
CPE (100% ethanol)	40	3.66	9.2

**Table 2:** Content of quercetin, myricetin, isorhamnetin, chlorogenic acid, caffeic acid, p-coumaric acid, total flavonols, total anthocyanins, total triterpenoids, and total proanthocyanidins in the six pomace extracts (mg/g of extract ± SD).

Average Analyte Concentration (mg/g of Extract)	CPAMFAH_2_O	CPMFAH_2_O	CPEFAH_2_O	CPM	CPMON	CPE
Quercetin	2.74 ± 0.01	3.09 ± 0.04	4.58 ± 0.01	2.35 ± 0.06	3.36 ± 0.05	2.20 ± 0.001
Myricetin	0.85 ± 0.002	1.01 ± 0.01	1.21 ± 0.001	0.53 ± 0.01	0.77 ± 0.01	0.50 ± 0.001
Isorhamnetin	0.44 ± 0.001	0.48 ± 0.03	0.76 ± 0.01	0.4 ± 0.01	0.53 ± 0.01	0.38 ± 0.001
Total flavonol aglycones	4.04 ± 0.01	4.57 ± 0.04	6.56 ± 0.003	3.29 ± 0.09	4.66 ± 0.07	3.09 ± 0.001
Total flavonol glycosides	2.81 ± 0.03	4.54 ± 0.18	5.12 ± 0.03	1.68 ± 0.05	3.04 ± 0.66	1.00 ± 0.02
**Total Flavonols**	**6.85 ± 0.03 ^d^**	**9.11 ± 0.16 ^b^**	**11.7 ± 0.03 ^a^**	**4.97 ± 0.14 ^e^**	**7.70 ± 0.13 ^c^**	**4.09 ± 0.02 ^f^**
Chlorogenic Acid	0.12 ± 0.004	0.14 ± 0.01	0.13 ± 0.01	0.05 ± 0.001	0.09 ± 0.01	0.05 ± 0.01
Caffeic Acid	0.26 ± 0.01	0.31 ± 0.02	0.27 ± 0.01	0.16 ± 0.003	0.23 ± 0.003	0.14 ± 0.001
P-Coumaric Acid	1.06 ± 0.02	1.67 ± 0.17	1.66 ± 0.03	0.70 ± 0.03	1.28 ± 0.02	0.73 ± 0.002
**Total Anthocyanins**	**1.33 ± 0.02 ^bc^**	**1.77 ± 0.13 ^a^**	**0.82 ± 0.01 ^d^**	**0.89 ± 0.01 ^c^**	**1.39 ± 0.01 ^ab^**	**0.42 ± 0.001 ^e^**
Ursolic Acid (UA)	44.7 ± 0.5	21.8 ± 0.3	70.3 ± 0.3	113 ± 1.2	116 ± 1.6	122 ± 1.8
Oleanolic Acid (OA)	36.7 ± 0.7	23.3 ± 0.9	53.6 ± 0.7	57.5 ± 1.4	65.8 ± 1.6	58.4 ±0.1
Trans-HCA-UA Ester	37.5 ± 1.1	11.9 ± 0.4	44.2 ± 0.9	39.4 ± 0.9	44.5 ± 2.3	40.3 ± 2.5
Cis-HCA-UA Ester	35.5 ± 1.2	16.0 ± 0.02	41.3 ± 2.2	33.6 ± 1.5	40.4 ± 1.7	31.9 ± 0.8
Corosolic Acid (CA)	6.94 ± 0.2	7.35 ± 0.1	12.0 ± 0.2	11.1 ± 0.2	13.0 ± 0.9	10.4 ± 0.2
Maslinic Acid (MA)	2.62 ± 0.2	4.64 ± 0.1	4.21 ± 0.1	2.73 ± 0.1	3.39 ± 0.1	2.51 ± 0.1
Cis-HCA-OA Ester	3.21 ± 0.1	0.64 ± 0.5	3.30 ± 1.9	2.99 ± 0.5	2.27 ± 1.7	2.77 ± 0.3
Trans-HCA-OA Ester	0.67 ± 1.1	0.00 ± 0.0	1.38 ± 0.1	1.28 ± 1.1	1.75 ± 2.0	0.00 ± 0.0
Total triterpenoid acids	90.9 ± 1.6	57.1 ± 1.4	140 ± 1.4	185 ± 2.9	199 ± 4.2	193 ± 2.1
Total triterpenoid HCA ^3^ esters	76.9 ± 3.5	28.5 ± 1.0	90.2 ± 5.1	77.4 ± 3.8	89.0 ± 7.6	75.0 ± 3.6
**Total Triterpenoids**	**168 ± 5.1 ^d^**	**85.6 ± 2.3 ^e^**	**230 ± 6.4 ^c^**	**262 ± 6.8 ^b^**	**287 ± 12 ^a^**	**268 ± 5.8 ^b^**
**Total Phenolic Content ^1^**	**166 ± 8.6 ^a^**	**144 ± 5.7 ^b^**	**71.7 ± 3.5 ^c^**	**54.3 ± 2.5 ^d^**	**59.6 ± 2.4 ^cd^**	**21.0 ± 1.5 ^e^**
**Total Proanthocyanidins ^2^**	**240 ± 38 ^a^**	**175 ± 7.9 ^b^**	**68.4 ± 4.3 ^c^**	**49.6 ± 4.3 ^d^**	**46.1 ± 4.7 ^d^**	**6.00 ± 0.8 ^e^**

Values are presented as means ± SD; n = 3, except for total flavonols, total anthocyanins, and phenolic acids, which were analyzed with n = 4. Different superscript letters within the same row indicate significant differences between solvent systems. One-way ANOVA followed by Tukey’s multiple comparisons test was used when homogeneity of variance was met; Brown–Forsythe–Welch ANOVA followed by Dunnett’s T3 test was used when variances were unequal. Significance was determined using multiplicity-adjusted *p* values, *p* < 0.05. ^1^ Total phenolic content is reported as mg gallic acid equivalents (GAE)/g of extract. ^2^ Total proanthocyanidins are reported as mg PAC equivalents/g of extract based on the modified DMAC assay. ^3^ HCA = p-hydroxycinnamoyl.

**Table 3:** IC_50_ for cranberry pomace extracts measured by DPPH, FRAP, and ABTS•+ assays.

Sample	DPPH IC_50_ (µg/mL)	FRAP IC_50_ (µg/mL)	ABTS•+ IC_50_ (µg/mL)
CPAMFAH_2_O	6.46 ± 2.8	2.61 ± 0.1	3.70 ± 0.6
CPMFAH_2_O	12.1 ± 1.7	4.09 ± 0.1	8.95 ± 1.0
CPEFAH_2_O	26.4 ± 5.5	6.66 ± 0.2	10.7 ± 1.5
CPMON	38.8 ± 2.9	9.55 ± 0.5	24.1 ± 5.7
CPM	49.5 ± 5.6	8.29 ± 0.1	28.0 ± 4.8
CPE	78.4 ± 6.8	14.9 ± 0.5	52.0 ± 9.0

Values are presented as means ± SD in µg/mL. Lower IC_50_ values indicate higher antioxidant activity.

**Table 4:** Principal component loading matrix for phytochemical and antioxidant activity parameters.

PC1	PC2	PC3	PC4	PC5	PC6	Variable
1.182	−0.384	−0.098	−0.170	−0.104	−0.795	DPPH
1.162	−0.056	−0.254	−0.175	0.644	−0.087	FRAP
1.150	0.791	−0.143	0.028	−0.357	0.047	TPC
1.138	−0.767	−0.217	−0.216	0.223	0.545	ABTS
1.100	1.023	−0.299	0.024	−0.468	0.213	Total PACs
−0.999	−0.783	−0.282	−0.780	−0.302	0.019	Total Triterpenoids
0.970	0.276	0.824	−0.424	−0.054	0.106	Total Anthocyanins
0.697	−2.429	0.102	0.322	−0.291	0.067	Total Flavonols

**Table 5:** Pearson correlations between mean antioxidant activity and phytochemical variables in cranberry pomace extracts.

Variable	Correlation *	*p*-Value
TPC	0.899	0.015
DPPH	0.998	0.000
FRAP	0.979	0.001
ABTS	0.994	0.000
Total Proanthocyanidins	0.857	0.029
Total Flavonols	0.665	0.150
Total Anthocyanins	0.748	0.087
Total Triterpenoids	−0.705	0.118

* Correlation coefficients represent Pearson r values. Mean antioxidant activity was calculated from inverse IC_50_ values for DPPH, FRAP, and ABTS•+.

**Table 6:** Pairwise Pearson correlation coefficients for phytochemical variables and antioxidant activity parameters in cranberry pomace extracts.

TPC	DPPH	FRAP	ABTS	Total PACs	Total Flavonols	Total Anthocyanins	Total Triterpenoids	Variable
1	0.918	0.924	0.852	0.991	0.358	0.746	−0.847	TPC
0.918	1	0.973	0.985	0.875	0.649	0.777	−0.731	DPPH
0.924	0.973	1	0.968	0.895	0.536	0.719	−0.738	FRAP
0.852	0.985	0.968	1	0.807	0.710	0.702	−0.649	ABTS
0.991	0.875	0.895	0.807	1	0.270	0.668	−0.811	Total PACs
0.358	0.649	0.536	0.710	0.270	1	0.389	−0.375	Total Flavonols
0.746	0.777	0.719	0.702	0.668	0.389	1	−0.678	Total Anthocyanins
−0.847	−0.731	−0.738	−0.649	−0.811	−0.375	−0.678	1	Total Triterpenoids

DPPH, ABTS, and FRAP data are based on inverse IC_50_ values: higher values indicate stronger antioxidant activity.

## Data Availability

The original contributions presented in this study are included in the article/Supplementary Material. Further inquiries can be directed to the corresponding author.

## Supplementary Materials

The following supporting information can be downloaded at https://www.mdpi.com/article/10.3390/antiox15060682/s1. Table S1: Validation parameters for HPLC-DAD analyses used for phytochemical quantification in cranberry pomace extracts; Figure S1: Total flavonols and total anthocyanins content in pomace extracts based on HPLC analysis; Figure S2: HPLC chromatogram of cranberry pomace acetone/methanol/formic acid/water (40/40/1/19) extract at 10 mg/mL, detected at 520 nm; Table S2: Anthocyanin content of CPAMFAH_2_O extract (mg/g extract) determined by HPLC-DAD (average ± SD, n = 4); Figure S3: HPLC chromatogram of cranberry pomace acetone/methanol/formic acid/water (40/40/1/19) extract at 10 mg/mL, detected at 355 nm; Table S3: Flavonol content of CPAMFAH_2_O extract (mg/g extract) as determined by HPLC-DAD (average ± SD, n = 4); Figure S4: HPLC chromatogram of cranberry pomace acetone/methanol/formic acid/water (40/40/1/19) extract at 10 mg/mL, detected at 310 nm. Chlorogenic acid retention time (RT) = 9.7 min, caffeic acid RT = 11.3 min, and p-coumaric acid RT = 13.9 min; Figure S5: Total phenolic content of the pomace extracts (n = 3).

## Abbreviations

The following abbreviations are used in this manuscript.

DPPH	(2,2-diphenyl-1-picrylhydrazyl)
ABTS•+	2,2′-azinobis(3-ethylbenzthiazolin-6-sulfonic acid)
FRAP	ferric reducing antioxidant power
DMAC	4-dimethylaminocinnamaldehyde
HPLC-DAD	high-performance liquid chromatography with diode array detector
UPLC-MS	ultrahigh-performance liquid chromatography–mass spectrometry
Q-TOF	quadrupole time of flight
ESI-MS	electrospray ionization–mass spectrometry
BEH	ethylene-bridged hybrid
CPAMFAH_2_O	cranberry pomace acetone–methanol–formic acid–water (40:40:1:19)
CPMFAH_2_O	cranberry pomace methanol–formic acid–water (70:0.1:29.9)
CPEFAH_2_O	cranberry pomace ethanol–formic acid–water (70:0.1:29.9)
CPMON	cranberry pomace methanol overnight (100%)
CPE	cranberry pomace ethanol (100%)
CPM	cranberry pomace methanol (100%)
