# Supplementary material for: Antioxidant Recovery from Massachusetts Cranberry Pomace: The Role of Solvent
Source: Antioxidants (Basel). 2026 May 29;15(6):682. doi: 10.3390/antiox15060682 (PMC13295805; doi:10.3390/antiox15060682)
Supplement: Supplementary file 1 [file antioxidants-15-00682-s001.zip › antioxidants-4282156-supplementary-nohigh.pdf]

Table S1: Validation parameters for HPLC-DAD analyses used for phytochemical quantification in cranberry pomace extracts.

Analyte	Linear Range (ppm)	Regression Equation	R^2	LOD (ppm)	LOQ (ppm)	Precision Range (%RSD)	Precision Average (%RSD)
Quercetin	3.12-100	y =175281x - 23816	1	0.010	0.031	0.15 - 1.80	0.43
Myricetin	3.12-100	y =137208x - 43126	1	0.003	0.009	0.02 - 1.70	0.70
Cyanidin-3-O-galactoside chloride	3.12-50	y =165774x - 29091	0.9998	0.210	0.637	0.05 - 3.14	0.84
Peonidin-3-O-galactoside chloride	6.25-100	y =14056x + 1787.2	1	0.030	0.090	0.06 - 2.18	0.77
p-Coumaric Acid	3.12-100	y =141618x + 6699.8	0.9999	0.009	0.027	0.10 - 2.33	1.45
Procyanidin A2	3.12-100	y =11263x - 3931.7	1	0.010	0.032	0.05 - 2.42	1.00
Procyanidin B2	3.12-50	y =13556x + 224.3	0.999	0.019	0.056	0.41 - 1.84	0.95

*LOD = limit of detection; LOQ = limit of quantification; %RSD = relative standard deviation; PPM =parts per million.

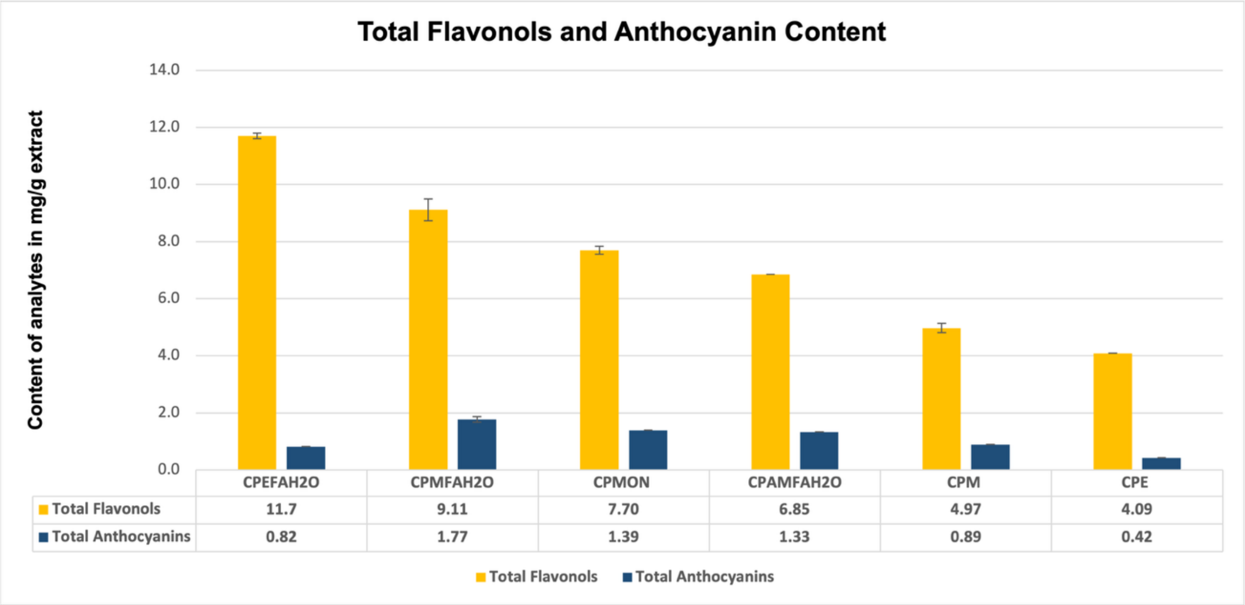

Figure S1. Total flavonols and total anthocyanins content in pomace extracts based on HPLC analysis.

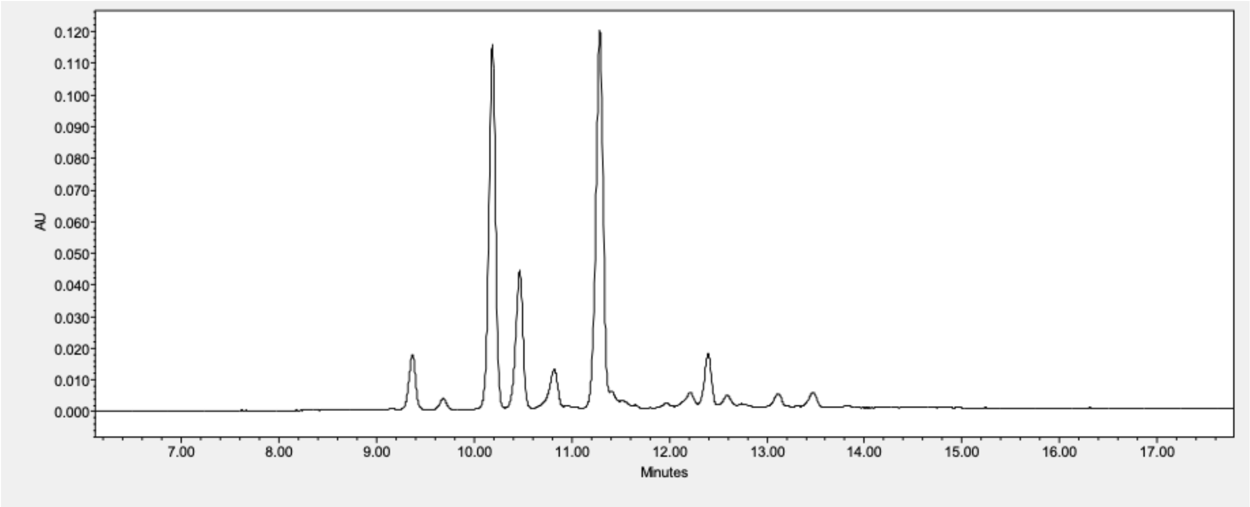

Figure S2. HPLC chromatogram of cranberry pomace acetone/methanol/formic acid/water (40/40/1/19) extract at 10 mg/mL, detected at 520 nm. Identified analyte peaks are listed in Table S2.

Table S2. Anthocyanin content of CPAMFAH_2O extract (mg/g extract) as determined by HPLC-DAD (average ± SD, n = 4).

Peak # at A520 nm	Retention Time (Min)	Anthocyanins	Concentration (mg/g)
1	9.4	Cyanidin-3-O-galactoside	0.08 ± 0.016
2	9.7	Cyanidin-3-O-glucoside	0.03 ±0.004
3	10.2	Cyanidin-3-O-arabinoside	0.35 ± 0.002
4	10.5	Peonidin 3-O-galactoside	0.15 ± 0.002
5	10.8	Peonidin-3-O-glucoside	0.07 ± 0.003
6	11.3	Peonidin-3-O-arabinoside	0.43 ± 0.002
7	12.2	Unidentified	0.04 ± 0.001
8	12.4	Unidentified	0.07 ± 0.001
9	12.6	Unidentified	0.03 ± 0.001
10	13.1	Unidentified	0.04 ± 0.001
11	13.5	Unidentified	0.04 ± 0.001
**Total**		**Total Anthocyanins**	**1.33 ± 0.02**

*Anthocyanin data expressed as equivalents of cyanidin-3-O-galactoside chloride.

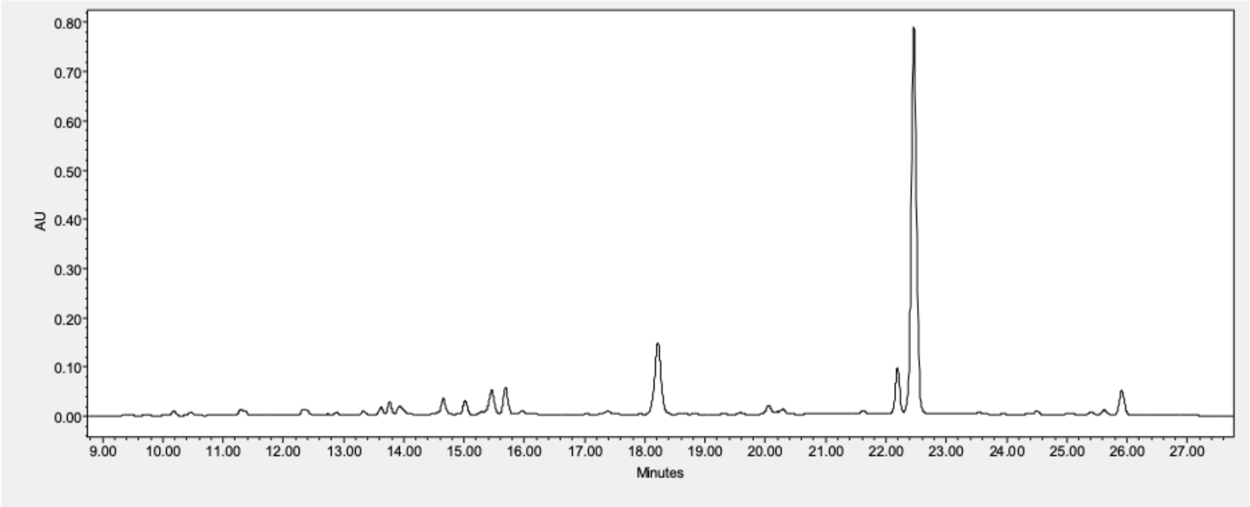

Figure S3. HPLC chromatogram of cranberry pomace acetone/methanol/formic acid/water (40/40/1/19) extract at 10 mg/mL, detected at 355 nm. Identified analyte peaks are listed in Table S3.

Table S3. Flavonol content of CPAMFAH_2O extract (mg/g extract) as determined by HPLC-DAD (average ± SD, n = 4).

**Peak # at A355 nm**	**Retention Time (Min)**	**Flavonol**	**Concentration (mg/g)**
1	13.3	Myricetin 3-xyloside	0.04 ± 0.004
2	13.6	Myricetin 3-arabinoside	0.06 ± 0.002
3	13.7	Quercetin 3-galactoside	0.16 ± 0.002
4	14.7	Quercetin 3-xyloside	0.33 ± 0.006
5	15.0	Quercetin 3-arabinopyranoside	0.23 ± 0.006
6	15.4	Quercetin 3-arabinofuranoside	0.59 ± 0.007
7	15.7	Quercetin 3-rhamnoside	0.51 ± 0.005
8	18.2	Myricetin	0.85 ± 0.002
9	20.3	Unidentified	0.10 ± 0.001
10	21.6	Unidentified	< LOQ
11	22.2	Unidentified	0.77 ± 0.002
12	22.5	Quercetin	2.74 ± 0.007
13	25.6	Unidentified	0.03 ± 0.001
14	25.9	Isorhamnetin	0.44 ± 0.001
**Total**		**Total Flavonols**	**6.85 ± 0.03**

*Flavonol data expressed as equivalents of quercetin-3-O-galactoside.

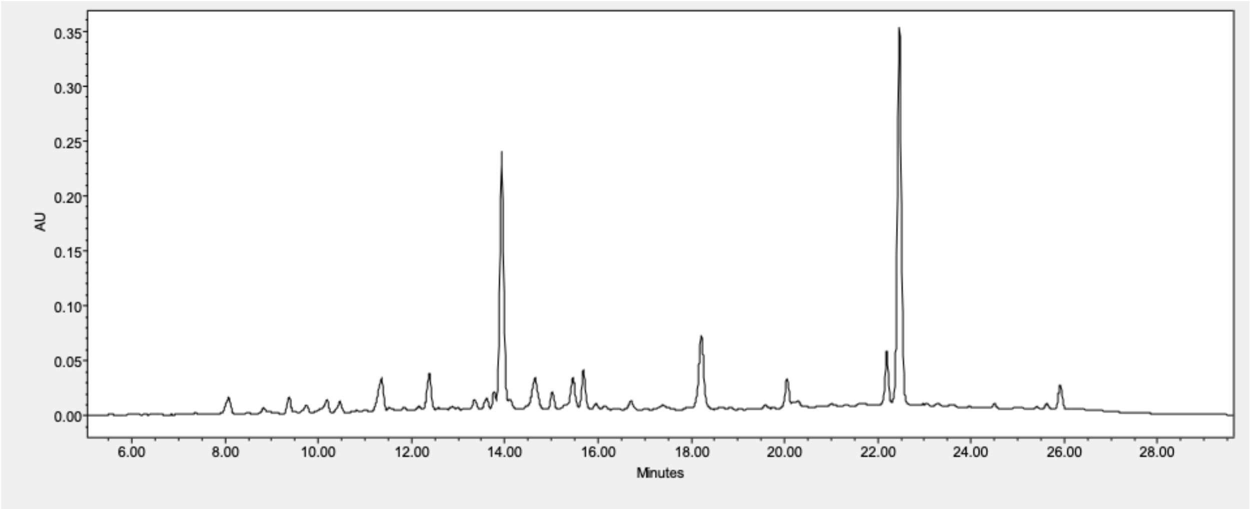

Figure S4. HPLC chromatogram of cranberry pomace acetone/methanol/formic acid/water (40/40/1/19) extract at 10 mg/mL, detected at 310 nm. Chlorogenic acid retention time (RT) = 9.7 min, caffeic acid RT = 11.3 min, and p-coumaric acid RT = 13.9 min.

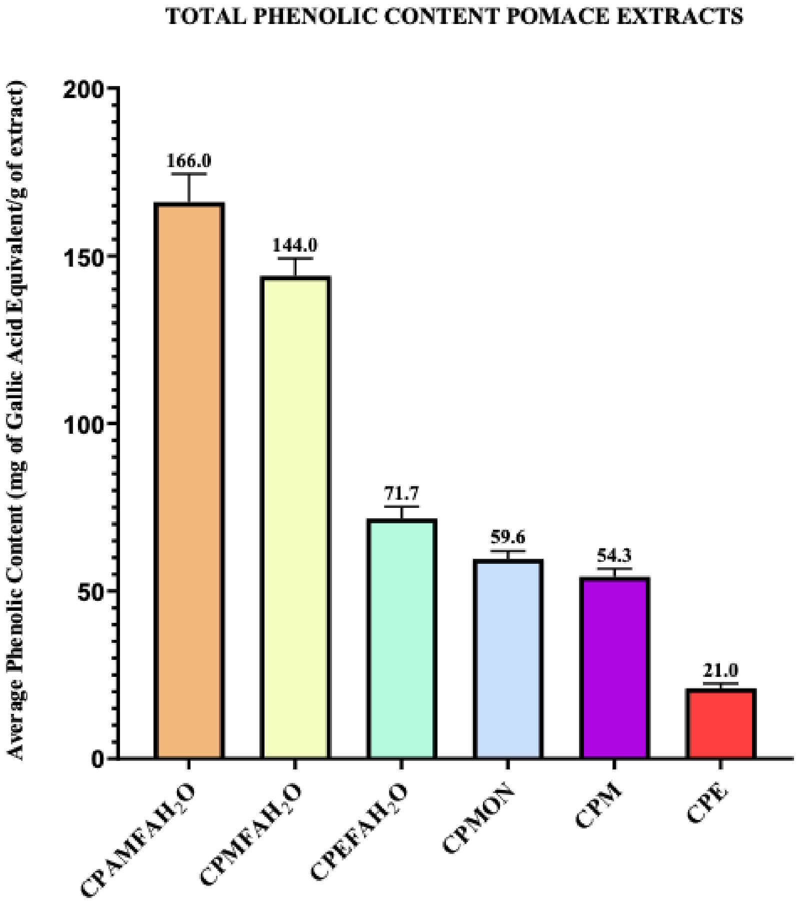

Figure S5. Total phenolic content of the pomace extracts (n=3)
